# Efficacy of liposome bupivacaine in transversus abdominis plane blocks for postoperative analgesia: a systematic review and meta-analysis

**DOI:** 10.3389/fmed.2026.1803767

**Published:** 2026-05-19

**Authors:** Qian Cao, Rong-Mu Lin, Yan Liu, Hong-Liang Liu

**Affiliations:** 1 Department of Anesthesia, Dazhou Dachuan District People’s Hospital (Dazhou Third People’s Hospital), Dazhou, Sichuan, China; 2 Department of Anesthesia, Zhuhai People’s Hospital (The Affiliated Hospital of Beijing Institute of Technology, Zhuhai Clinical Medical College of Jinan University), Zhuhai, Guangdong, China

**Keywords:** liposomal bupivacaine, meta-analysis, postoperative analgesia, standard bupivacaine, transversus abdominis plane block

## Abstract

**Objective:**

This meta-analysis evaluates the efficacy of liposomal bupivacaine (LB) versus standard bupivacaine (SB), with or without adjuvants, in transversus abdominis plane (TAP) blocks for postoperative analgesia.

**Methods:**

Registered in PROSPERO (CRD420251084761), this meta-analysis screened randomized controlled trials (RCTs) published from 2000 to 2025 in PubMed, the Cochrane Library, Embase, and Wanfang databases. The primary outcome was the cumulative morphine milligram equivalent (MME) within 1 day after surgery. Secondary outcomes included the MME within 2–3 days after surgery, pain scores on postoperative days 1–3, time to first rescue analgesia, time to first flatus passage, time to first ambulation, postoperative nausea and vomiting (PONV), local anesthetic-related adverse events and toxicity, and length of hospital stay.

**Results:**

A total of 13 randomized controlled trials (RCTs) (*n* = 1,528) were included in this meta-analysis. Compared to standard bupivacaine, liposomal bupivacaine reduced the MME within 1 day after surgery (mean difference (MD): −0.62 mg, 95%CI: −1.13 to −0.11). Liposomal bupivacaine also reduced pain scores on postoperative days 1–3, measured by the numeric rating scale (NRS; day 1: MD −0.65, 95% CI −1.03 to −0.28; day 2: MD −0.40, 95% CI −0.77 to −0.04; day 3: MD −0.30, 95% CI -0.48 to −0.13), prolonged the duration of analgesia (MD 5.39 h, 95% CI 0.06–10.72), and accelerated flatus passage (MD −0.23 day, 95% CI −0.35 to −0.11). No superiority of LB over SB plus adjuvants was observed in postoperative opioid consumption or pain relief. The quality of evidence was rated as low to very low for these outcomes.

**Conclusion:**

Liposomal bupivacaine used in TAP blocks can provide only minimal reductions in opioid consumption and pain relief after surgery compared to standard bupivacaine. Given that the quality of evidence was low to very low, large-scale RCTs are required to validate our findings.

## Introduction

The transversus abdominis plane (TAP) block delivers local anesthetic into the neurovascular plane between the internal oblique and transversus abdominis muscles and has become an important component of multimodal analgesia for postoperative pain control in abdominal surgical procedures (e.g., cesarean section, hysterectomy, abdominal wall surgery, and rectal resection). It has been shown to be more cost-effective compared to epidural blocks (1). Standard bupivacaine (SB), a long-acting local anesthetic, is commonly used in TAP blocks but provides analgesia for less than 12 h (2), limiting its clinical utility.

A 2016 Cochrane review confirmed the efficacy of liposomal bupivacaine (LB) in peripheral nerve blocks (3), which overcomes conventional limitations by capturing bupivacaine in vesicles for slow tissue release, providing analgesia for 72 h (4). Consequently, LB has gained interest for its potential to prolong block duration. Several studies have demonstrated that adding adjuvants to SB (e.g., adrenaline, morphine, dexmedetomidine, or dexamethasone) in TAP blocks can improve postoperative analgesia and reduce opioid consumption by extending the analgesic effect of SB by several hours, sometimes up to 18–20 h (5–7). Therefore, LB can theoretically provide longer analgesia and reduce morphine consumption when used in TAP blocks compared to SB, with or without adjuvants, and has become a research focus over the past few years.

This systematic review and meta-analysis pooled trials comparing TAP blocks using LB with those using SB or SB plus adjuvants and evaluated the efficacy of LB in TAP blocks.

## Methods

This systematic review and meta-analysis was conducted in accordance with the Preferred Reporting Items for Systematic Reviews and Meta-analyses (PRISMA) guidelines and registered in PROSPERO (Registration No: CRD420251084761).

### Literature search

A comprehensive search was performed in PubMed, the Cochrane Library, Embase, and Wanfang databases from January 2000 to July 2025. The search terms included liposome bupivacaine, liposomal bupivacaine, bupivacaine liposome, bupivacaine, conventional bupivacaine, standard bupivacaine, plane bupivacaine, simple bupivacaine, regular bupivacaine, standardized bupivacaine, transversus abdominis plane block, TAP block, clinical trials, and randomized controlled trials (RCTs). The detailed search strategy for PubMed is provided in Supplementary file 1. Conference abstracts were screened for eligibility if sufficient data were available. In addition, the reference lists of the retrieved articles were manually reviewed to identify potential trials.

### Inclusion and exclusion criteria

The inclusion criteria included the following: (1) studies evaluating TAP blocks using LB or SB for postoperative analgesia; (2) studies reporting comparisons between LB and SB, LB plus SB and SB, or LB versus SB plus adjuvants (injected locally into the TAP); (3) RCTs involving adult patients (≥18 years); and (4) no restrictions on publication language.

The exclusion criteria were as follows: (1) studies reporting TAP blocks using LB versus opioids or non-bupivacaine local anesthetics; (2) studies comparing TAP blocks using LB with other regional techniques (local incision infiltration, epidural anesthesia, or intrathecal block); (3) studies involving pediatric patients(<18 years); and (4) abstracts with insufficient data, editorials, or review articles.

### Outcomes

The primary outcome was the cumulative morphine milligram equivalent (MME) within 1 day after surgery, a metric used to convert each opioid dose into an equivalent amount of morphine based on its relative analgesic strength. Secondary outcomes included the cumulative MME within 2–3 days after surgery, time to first rescue analgesia, and postoperative average pain scores on days 1–3, assessed using the numeric rating scale (NRS), where 0 indicates no pain and 10 indicates the most severe pain. Other secondary outcomes were time to first passage of flatus, incidence of postoperative nausea and vomiting (PONV), local anesthetic-related adverse events or toxicity, and length of postoperative hospital stay.

### Data extraction and quality assessment

In total, two investigators independently searched the databases, screened the literature according to the inclusion and exclusion criteria, and extracted data, including first author, publication year, surgical type, group allocations, TAP methods, patients’ age, interventions, sample size, and outcome measures. Any discrepancies during screening or data extraction were resolved by consensus among all investigators. During data extraction, the following group definitions were applied: the LB group for TAP blocks with liposomal bupivacaine, the LB + B group for liposomal bupivacaine plus bupivacaine, the SB group for TAP blocks with standard bupivacaine alone, and the SB+ adjuvant group for standard bupivacaine plus adjuvants (e.g., dexamethasone or adrenaline).

The risk of bias was assessed by evaluating “adequate sequence generation,” “allocation concealment,” “blinding,” “incomplete outcome data addressed,” “free of selective reporting,” and “free of other bias,” as recommended by the Cochrane Collaboration. Publication bias was assessed using a funnel plot and Egger’s test, with a *p*-value of >0.05 indicating no significant bias.

The quality of the included studies was evaluated using the Jadad scoring system, which considers sample size calculation, generation of the allocation sequence, allocation concealment, methods of randomization, blinding, and descriptions of protocol deviations, withdrawals, and dropouts. Trials with a quality score of less than 3 were excluded. The Grading of Recommendations, Assessment, Development, and Evaluation (GRADE) methodology was used to appraise the overall evidence-based quality for the outcomes (8). Furthermore, two investigators independently assessed the risk of bias, the quality of the included studies, and the overall evidence using the GRADE, with any discrepancies resolved by consensus among all investigators.

### Statistical analysis

Review Manager version 5.2 for Windows (the Cochrane Collaboration, Oxford, UK) was used to assess the risk of bias of the included studies and to perform the meta-analysis. The STATA software (version 18.0, StataCorp, College Station, TX, USA) was used to perform meta-regression to assess the association between covariates (e.g., age, sample size, TAP methods, and LB concentration and volume) and the primary outcome. When data were presented as median (interquartile range or range), they were transformed into mean ± SD using the methods described by Wan et al. (9). When data were presented as graphs, they were transformed into numbers using the PlotDigitizer software. The effect size for continuous cumulative outcomes was presented as the mean difference (MD) with a 95% confidence interval (CI). The effect size for dichotomous outcomes was presented as the risk ratio (RR) with a 95%CI. Between-study heterogeneity was determined using the *I*^2^ statistic, and the levels of heterogeneity were defined as low (*I^2^* ≤ 25%), moderate (*I*^2^ = 25–50%), and high (*I*^2^ > 50%). In the case of *I*^2^ < 50%, a fixed effects model was used, and in the case of *I*^2^ ≥ 50%, a random effects model was used. Sensitivity analyses were conducted to assess the robustness of the results, meta-regression analysis was conducted to assess the relationship between covariates (sample size, age, LB concentration and volume, surgical type, TAP methods, and publication time) and the primary outcome, and subgroup analysis was performed when necessary.

## Results

### Characteristics of the eligible trials

**Figure 1:**
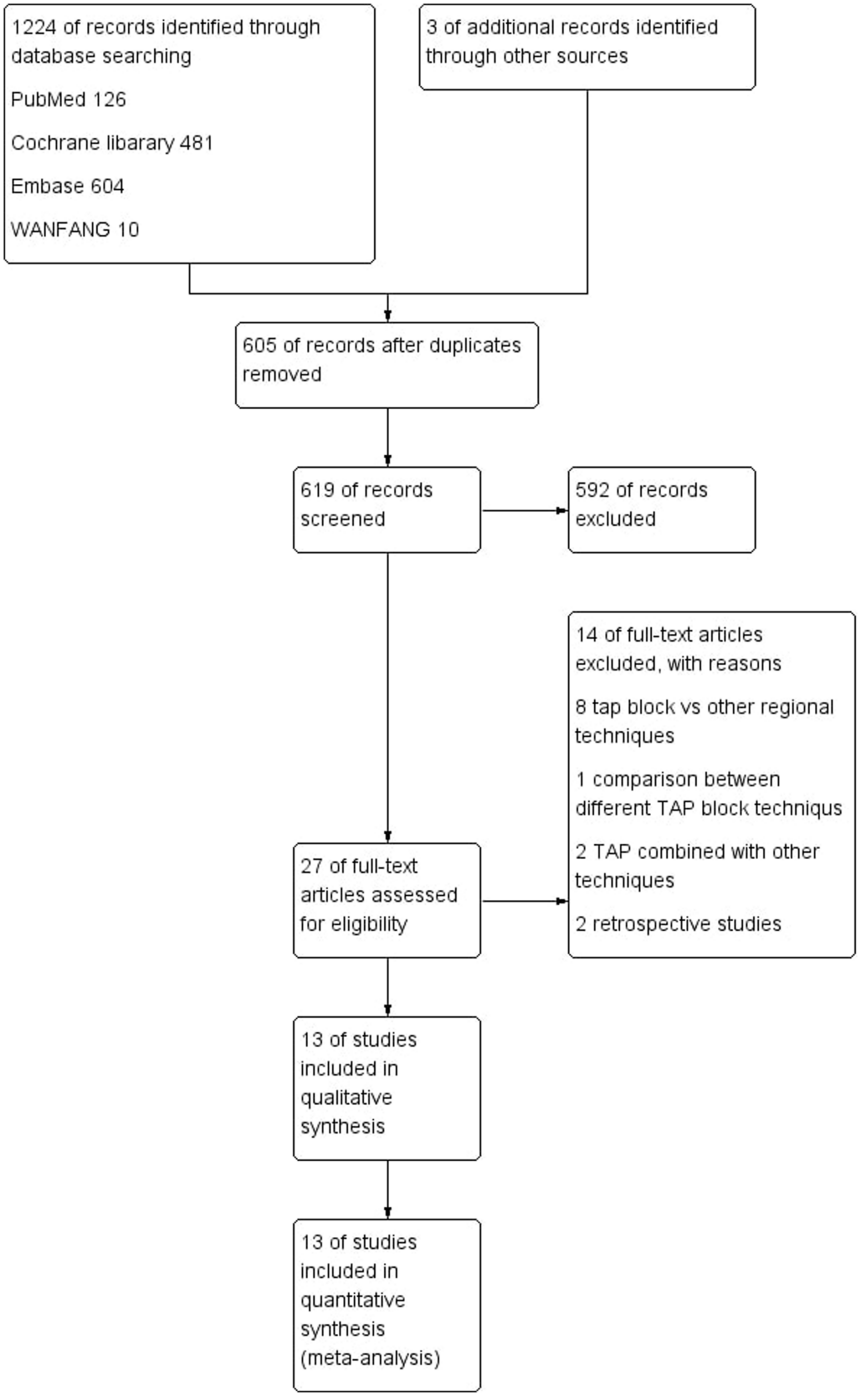
Flowchart of study selection.

**Figure 2:**
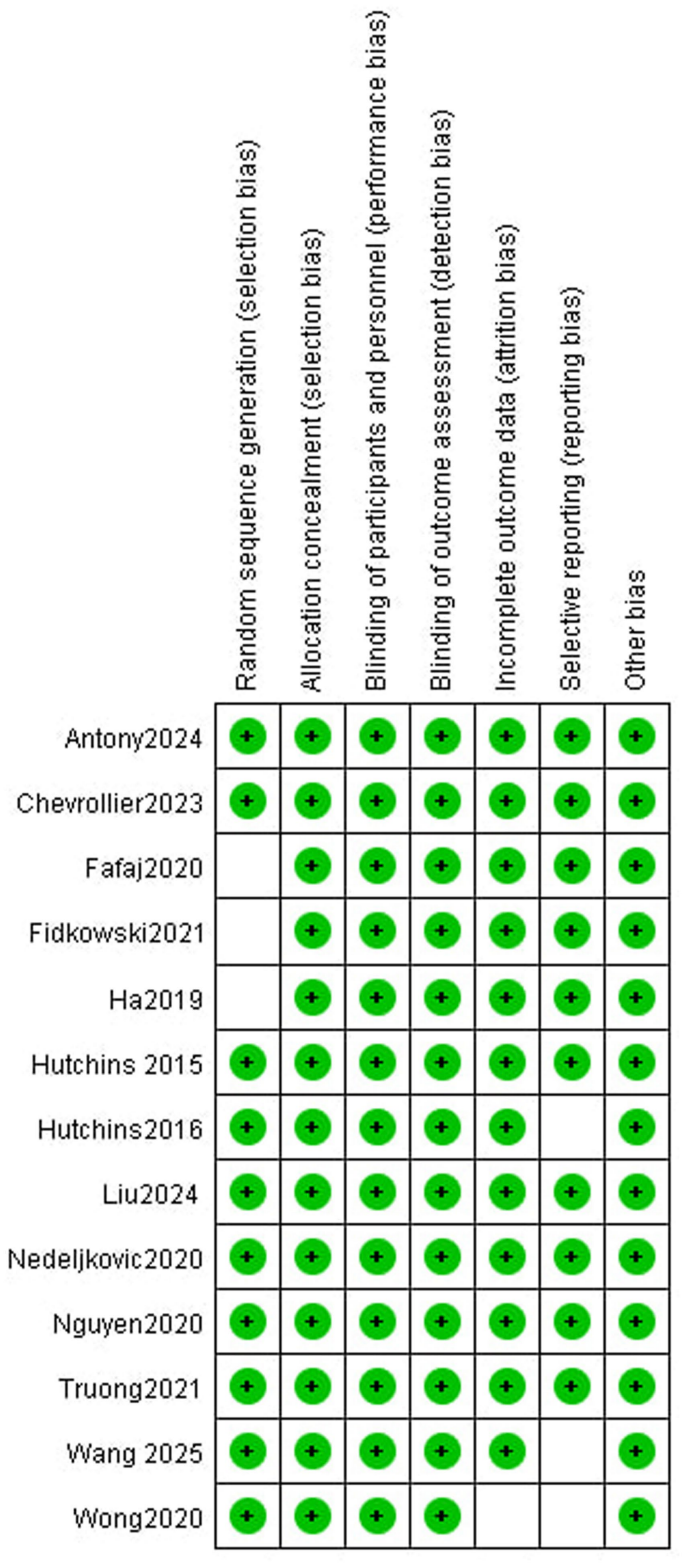
Risk of bias summary. Green indicates low risk of bias, red indicates high risk of bias, and white indicates unclear risk of bias.

A total of 1,224 articles were initially identified from the databases: 126 from PubMed, 481 from the Cochrane Library, 604 from Embase, 10 from Wanfang, and three from other sources. After removing duplicates, 619 articles remained for title and abstract screening. Subsequently, 592 articles were excluded, leaving 27 full-text articles for eligibility assessment. After excluding 14 additional articles, 13 RCTs involving 1,528 patients were included in the meta-analysis (10–22). Among these, 10 studies compared LB versus SB (10–19), whereas three compared LB versus SB plus adjuvants (20–22). The screening process is summarized in Figure 1, trial characteristics in Table 1, and the risk of bias assessment in Figure 2.

**Table 1:** Characteristics of the included studies.

Author	Year	Surgery	Groups and intervention (bilateral TAP block)	TAP methods	Sample size	Age	Outcomes	Jadad score
Liposomal bupivacaine versus standard bupivacaine								
Antony et al. (10)	2024	Cesarean section	LB: 1.3% LB 20 mL + 0.25% bup 30 mL + NS 30 mL	Laparoscopic-guided	*n* = 31	33.0 (3.8)	NRS, MME, time to first rescue or flatus passage, hospital stay	7
			SB: 0.25% bup 30 mL + NS 50 mL (40 mL per side)		*n* = 29	33.7 (5.3)		
Chevrollier et al. (11)	2023	Laparoscopic colorectal resection	LB: 1.3% LB 20 mL + NS 55 mL + 0.5% bup 25 mL	Laparoscopic-guided	*n* = 38	57.9 (16.5)	NRS, MME, hospital stay	5
					*n* = 38			
			SB: 0.5% bup 30 mL + NS 70 mL (50 mL per side)					
						60.6 (15.2)		
Fafaj et al. (12)	2022	Open hernia repair	LB: 1.3% LB 20 mL + 0.25% bup 60 mL + NS 40 mL	Laparoscopic-guided	*n* = 57	59.0 (52, 58)	NRS, MME, hospital stay	5
					*n* = 55	58.0 (53, 69)		
			SB: 0.25% bup 60 mL + NS 60 mL (60 mL per side)					
Fidkowski et al. (13)	2021	Open hysterectomy	LB: 1.3% LB 20 mL + NS 40 mL	Ultrasound-guided	*n* = 27	60.3 (13.0)	NRS, MME, time to first rescue, hospital stay	7
			LB + SB: 1.3% LB 20 mL + 0.25% bup 40 mL		*n* = 25	63.0 (10.9)		
			SB: 0.25% bup 60 mL (30 mL per side)		*n* = 25	59.8 (12.7)		
Ha et al. (14)	2019	Autologous microvascular breast reconstruction	LB: 0.89% LB 30 mL	Laparoscopic-guided	*n* = 22	49 (9.2)	NRS, MME, time to first ambulation, hospital stay	5
			B: 0.25% bupivacaine 30 mL (15 mL per side)		*n* = 22	49 (10)		
Liu et al. (15)	2024	Cesarean section	LB: 1.3% LB 20 mL + NS 20 mL	Ultrasound-guided	*n* = 49	31 (5.5)	NRS, MME, hospital stay, time to first rescue, flatus passage, or ambulation	7
			LB + SB: 1.3% LB 20 mL + 0.125% bup 20 mL		*n* = 49	32 (4.8)		
			SB: 0.125% bup 40 mL (20 mL per side)		*n* = 49	31 (4.9)		
Nedeljkovic et al. (16)	2020	Cesarean section	LB + SB: 1.3% LB 20 mL + 0.125% bup 40 mL	Ultrasound-guided	*n* = 97	34 (19, 47)	NRS, MME, time to first ambulation	7
			SB: 0.125% bup 40 mL + NS 20 mL(30 mL per side)					
					*n* = 89	33 (24, 44)		
Nguyen et al. (17)	2024	DIEP flap reconstruction	LB: 1.3% LB 20 mL + 0.25% bup 20 mL	Ultrasound-guided	*n* = 30	53.0 (9.5)	NRS, MME, time to first rescue, flatus passage, or ambulation, hospital stay	7
			SB: 0.25% bup 40 mL (20 mL per side)		*n* = 30	52.2 (9.8)		
Wong et al. (18)	2020	Weight loss surgery	LB: 1.3% LB 20 mL + 0.25% bup 30 ml + NS 100 mL	Laparoscopic-guided	*n* = 75	42.1 (9.8)	NRS, MME, hospital stay	5
					*n* = 73	39.4 (10.9)		
			SB: 0.25% bup 50 mL + NS 100 mL (75 mL per side)					
Wang et al. (19)	2025	Laparoscopic colorectal surgery	LB: 1.3% LB 20 mL + NS 20 mL	Ultrasound-guided	*n* = 200	72 (2)	Pain scores, hospital stay	5
			SB: 1% SB 10 mL + NS 30 mL (20 mL per side)		*n* = 200	71 (2)		
Liposomal bupivacaine versus standard bupivacaine plus adjuvants								
Hutchins et al. (20)	2016	Laparoscopic nephrectomy	LB: 1.3% LB 20 mL + NS 40 mL	Ultrasound-guided	*n* = 30	41.0 (12.5)	NRS, MME, hospital stay	5
			SB + AD: 0.25% bup 60 mL (30 mL per side)		*n* = 29	38.0 (12.6)		
Hutchins et al. (21)	2015	Laparoscopic hysterectomy	LB: 1.3% LB 20 mL + NS 40 mL	Ultrasound-guided	*n* = 28	60.5 (10.8)	NRS, MME	7
					*n* = 30	56.8 (10.0)		
			SB + AD: 0.25% bup 60 mL (AD1: 200,000) (30 mL per side)					
Truong et al. (22)	2021	Laparoscopic colorectal surgery	LB: 1.3% LB 20 mL + 0.5% bup (total volume: 1 mL/kg)	Laparoscopic-guided	*n* = 50	42 (29, 57)	NRS, MME, time to first flatus passage or ambulation, hospital stay	5
					*n* = 51	42 (29, 67)		
			SB + AD + Dex: 0.25% bup (AD1: 200,000 + Dex 8 mg) (total volume: 1 mL/kg) (half of 1 mL/kg per side)					

LB, liposomal bupivacaine; SB, bupivacaine; MME, morphine milligram equivalent; DIEP, deep inferior epigastric artery perforator; NRS, numeric rating scale.

Age was presented as mean (SD) or median (IQR).

### Meta-analysis

Fidkowski et al. (13) and Liu et al. (15) compared liposomal bupivacaine versus bupivacaine or liposomal bupivacaine plus bupivacaine versus bupivacaine. These studies were split into two substudies in the forest plots, labeled as Liu 2024 (1) and Liu 2024 (2), or Fidkowski 2021 (1) and Fidkowski 2021 (2).

### The primary outcome—cumulative MME within 1 day after surgery

#### LB versus SB

**Figure 3:**
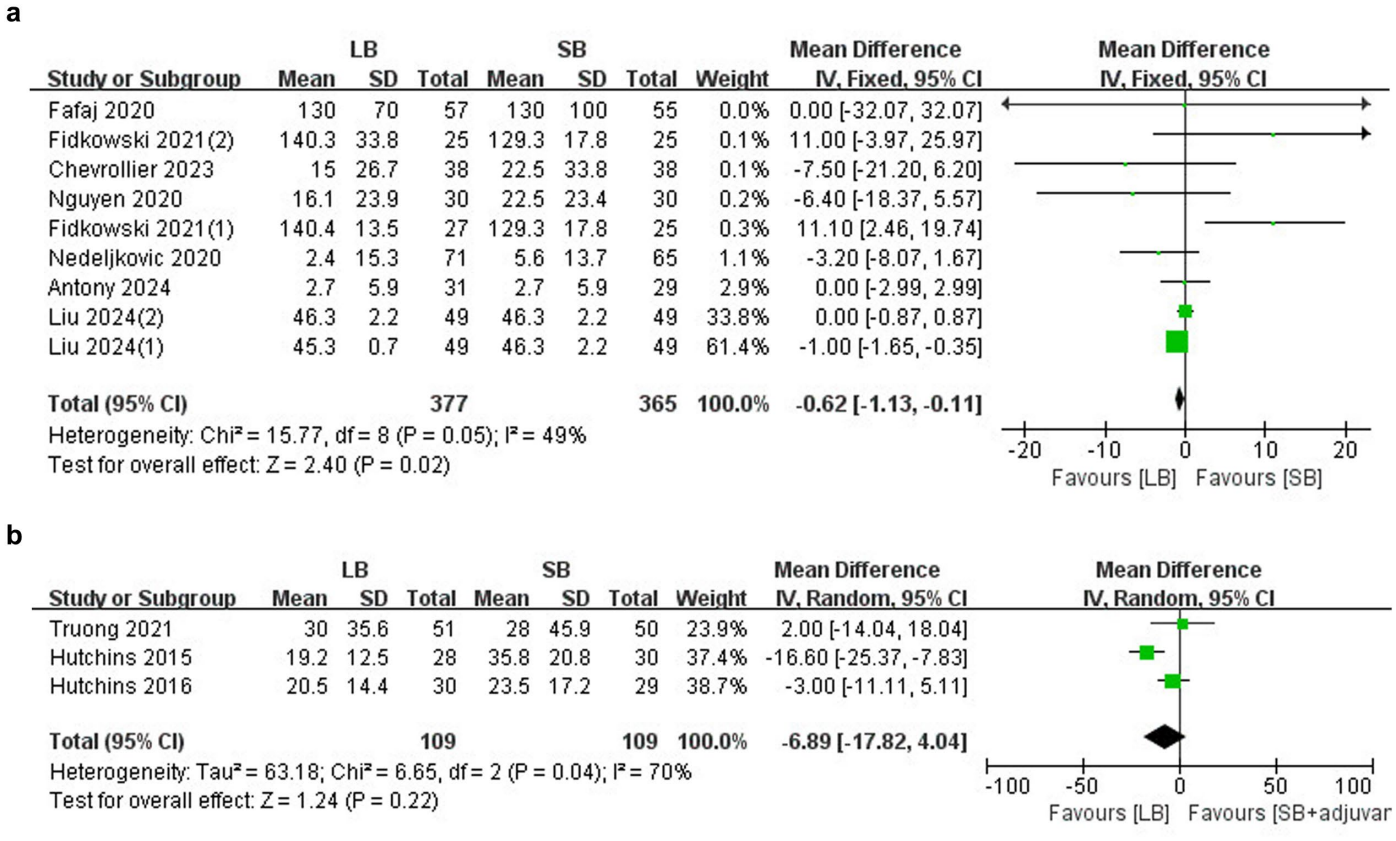
Forest plots of the cumulative MME within 1 day after surgery: **(a)** comparison between LB and SB; **(b)** comparison between LB and SB+ adjuvants.

A total of seven RCTs (*n* = 668) (10–13, 15–17) revealed that LB reduced the MME compared to SB (MD: −0.62 mg, 95%CI: −1.13 to −0.11, *p* = 0.02, *I*^2^ = 49%) (Figure 3a). No publication bias was detected based on the funnel plot and Egger’s test (*p* = 0.286). Sensitivity analysis showed no changes in the results when any individual study was omitted. Meta-regression was conducted to assess the relationship between covariates (sample size, age, LB concentration and volume, surgical type, TAP methods, and publication time) and the primary outcome, and it did not identify any of these covariates as a significant moderator (Supplementary file 2). As the TAP block was a critical factor in this meta-analysis, a subgroup analysis of different TAP methods was performed. The results showed that LB administered under either an ultrasound-guided TAP block (MD: −0.35 mg, 95%CI: −1.94 to 1.24, *p* = 0.66, *I*^2^ = 66%) or a laparoscopic-guided TAP block (MD: −0.34 mg, 95%CI: −3.24 to 2.57, *p* = 0.82, *I*^2^ = 0%) did not reduce the MME within 1 day after surgery compared to SB.

#### LB versus SB plus adjuvants

Only three trials (*n* = 318) (20–22) reported the cumulative MME within 1 day after surgery, and the results revealed no between-group differences (MD: −6.89 mg, 95%CI: −17.82-4.04, *p* = 0.22, *I*^2^ = 70%) (Figure 3b). Sensitivity analysis revealed no changes in the results when any individual study was omitted.

### Secondary outcomes

#### LB versus SB

**Figure 4:**
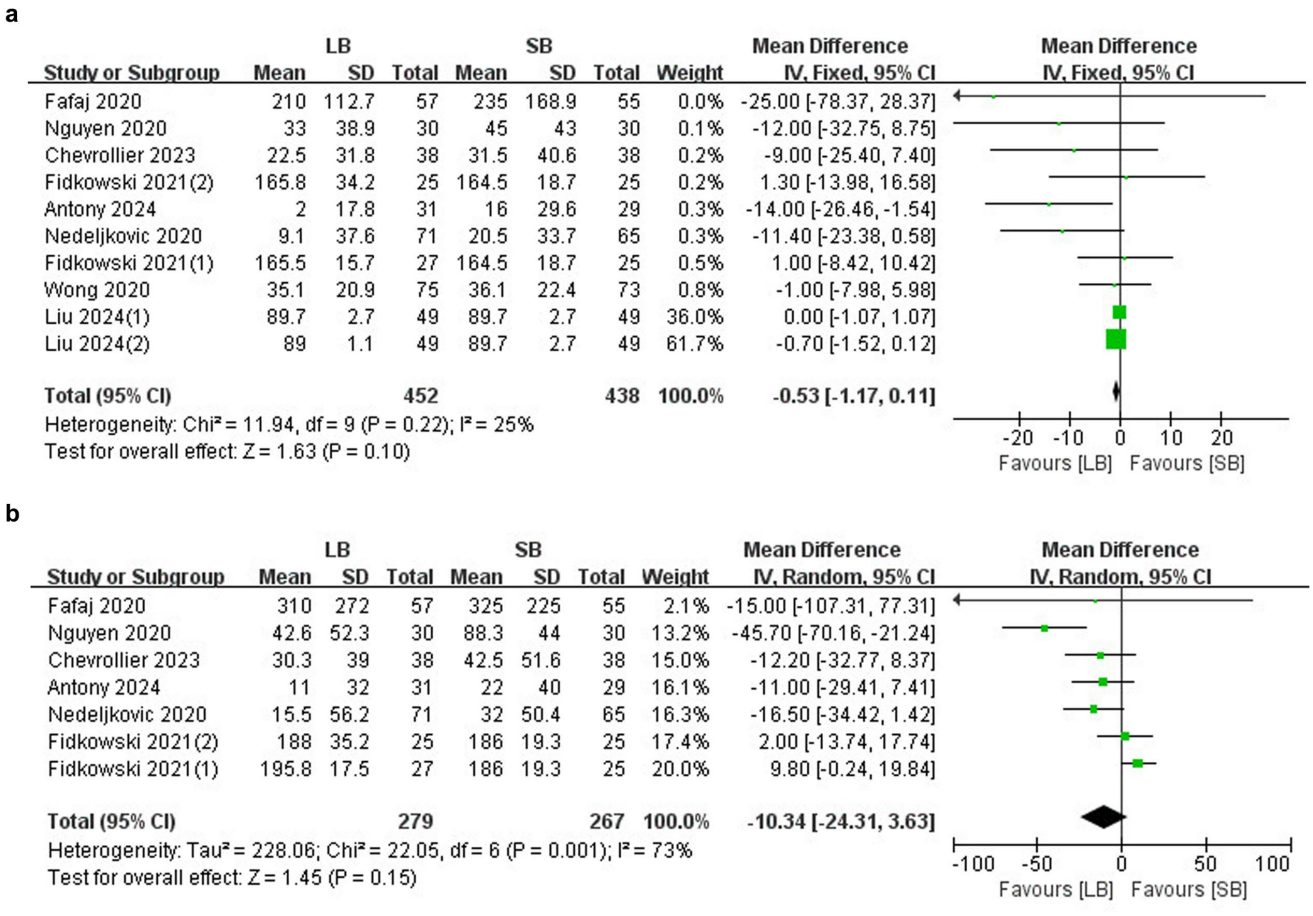
Forest plots of the cumulative MME comparing LB with SB. **(a)** MME within 2 days **(b)** MME within 3 days.

MME within 2–3 days after surgery: Eight RCTs (*n* = 816) (10–13, 15–18) showed no between-group differences in the MME within 2 days (MD: −0.53 mg, 95%CI: −1.17 to 0.11, *p* = 0.10, *I*^2^ = 25%) (Figure 4a). Subgroup analysis showed that LB administered under an ultrasound-guided TAP block did not reduce the MME compared to SB (MD −0.38 mg, 95%CI: −0.96 to 0.20, *p* = 0.19, *I*^2^ = 16%), nor did LB administered under a laparoscopic-guided TAP block (MD −4.39 mg, 95%CI: −10.61 to 0.75, *p* = 0.09, *I*^2^ = 0). A total of six RCTs (*n* = 521) (10–13, 16, 17) showed no between-group differences in the MME within 3 days (MD: −10.34 mg, 95%CI: −24.31 to 3.63, *p* = 0.15, *I*^2^ = 73%) (Figure 4b). Sensitivity analysis revealed that the result became statistically significant when the study by Fidkowski et al. (13) was omitted. Subgroup analysis revealed that LB administered under an ultrasound-guided TAP block did not reduce the MME compared to SB (MD −10.55 mg, 95%CI: −31.43 to 10.34, *p* = 0.32, *I*^2^ = 85%), nor did LB administered under a laparoscopic-guided TAP block (MD −11.61 mg, 95%CI: −25.18 to 1.96, *p* = 0.09, *I*^2^ = 0).

##### Average pain score

**Figure 5:**
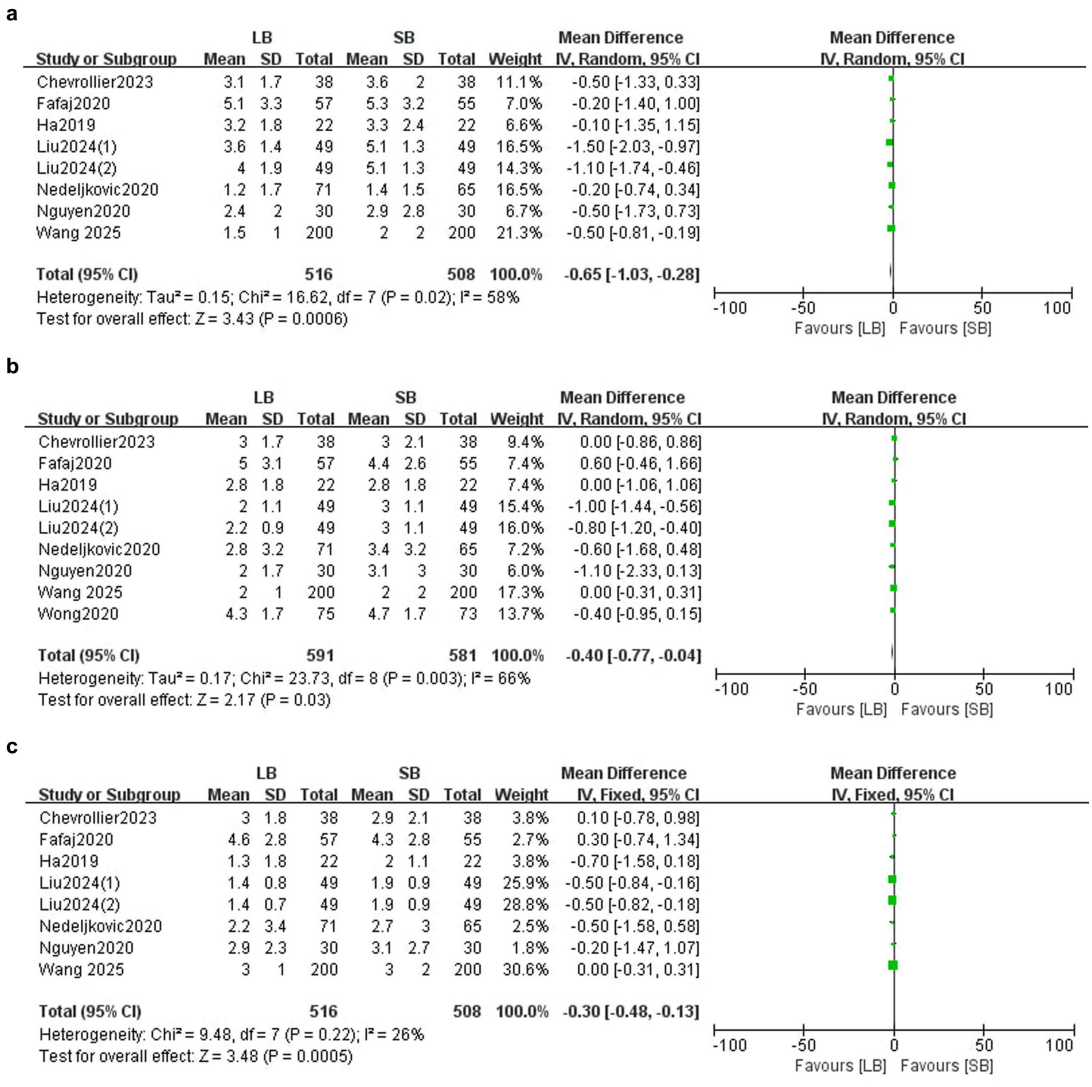
Forest plots of pain scores comparing LB with SB. **(a)** NRS on day 1; **(b)** NRS on day 2; **(c)** NRS on day 3.

Seven RCTs (*n* = 975) (11, 12, 14–17, 19), eight RCTs (*n* = 1,123) (11, 12, 14–19), and seven RCTs (*n* = 975) (11, 12, 14–17, 19) reported average pain scores on postoperative days 1, 2, and 3, respectively. LB significantly reduced the average pain score on day 1 (MD: −0.65, 95%CI: −1.03 to −0.28, *p* = 0.0006, *I*^2^ = 58%), day 2 (MD: −0.40, 95%CI: −0.77 to −0.04, *p* = 0.03, *I*^2^ = 66%), and day 3 (MD: −0.30, 95%CI: −0.48 to −0.13, *p* = 0.0005, *I*^2^ = 26%) (Figures 5a–c). Sensitivity analysis showed that the results were robust when any individual study was omitted on day 1, day 2, or day 3. Subgroup analyses revealed that, compared to SB, LB under an ultrasound-guided TAP block significantly reduced pain scores on days 1, 2, and 3 after surgery (MD: −0.77, 95%CI: −1.23 to −0.28, *p* = 0.002, *I*^2^ = 73%; MD: −0.64, 95%CI: −1.15 to −0.13, *p* = 0.01, *I*^2^ = 78%; MD: −0.32, 95%CI: −0.50 to −0.14, *p* = 0.0005, *I*^2^ = 39%), while LB under a laparoscopic-guided TAP block did not reduce pain scores on days 1, 2, and 3 (MD: −0.33, 95%CI: −0.93 to 0.27, *p* = 0.28, *I*^2^ = 0%; MD: −0.12, 95%CI: −0.52 to 0.27, *p* = 0.54, *I*^2^ = 0%; MD: −0.14, 95%CI: −0.67 to 0.39, *p* = 0.61, *I*^2^ = 21%).

##### Time to first rescue

Four RCTs (*n* = 344) (10, 13, 15, 17) showed that LB prolonged the time to first rescue (MD: 5.39 h, 95%CI: 0.06–10.72, *p* = 0.05, *I*^2^ = 78%) (Supplementary Figure 1).

##### Time to first passage of flatus

Three RCTs (*n* = 267) (10, 15, 17) showed that liposomal bupivacaine shortened the time to passage of flatus (MD: −0.23 day, 95%CI: −0.35 to −0.11, *p* = 0.0002, *I*^2^ = 81%) (Supplementary Figure 2).

##### Time to first ambulation

Five trials (*n* = 537) (14–18) revealed no between-group differences (MD: −0.03 day, 95%CI: −0.07–0.00, *p* = 0.08, *I*^2^ = 95%) (Supplementary Figure 3).

##### PONV

Five trials (*n* = 596) (10, 15–18) showed no between-group differences (RR: 0.67, 95%CI: 0.43–1.03, *p* = 0.07, *I*^2^ = 0%) (Supplementary Figure 4).

Local anesthetic-related toxicity or adverse events: Four trials (*n* = 543) (10, 15, 16, 18) revealed no between-group differences (RR: 0.84, 95%CI: 0.46–1.54, *p* = 0.57, *I*^2^ = 0%) (Supplementary Figure 5).

##### Hospital stay

Nine trials (*n* = 1,124) (10–15, 17–19) showed no between-group differences (MD: 0.11 day, 95%CI: −0.30–0.07, *p* = 0.23, *I*^2^ = 95%) (Supplementary Figure 6).

#### LB versus SB plus adjuvants

##### MME

**Figure 6:**
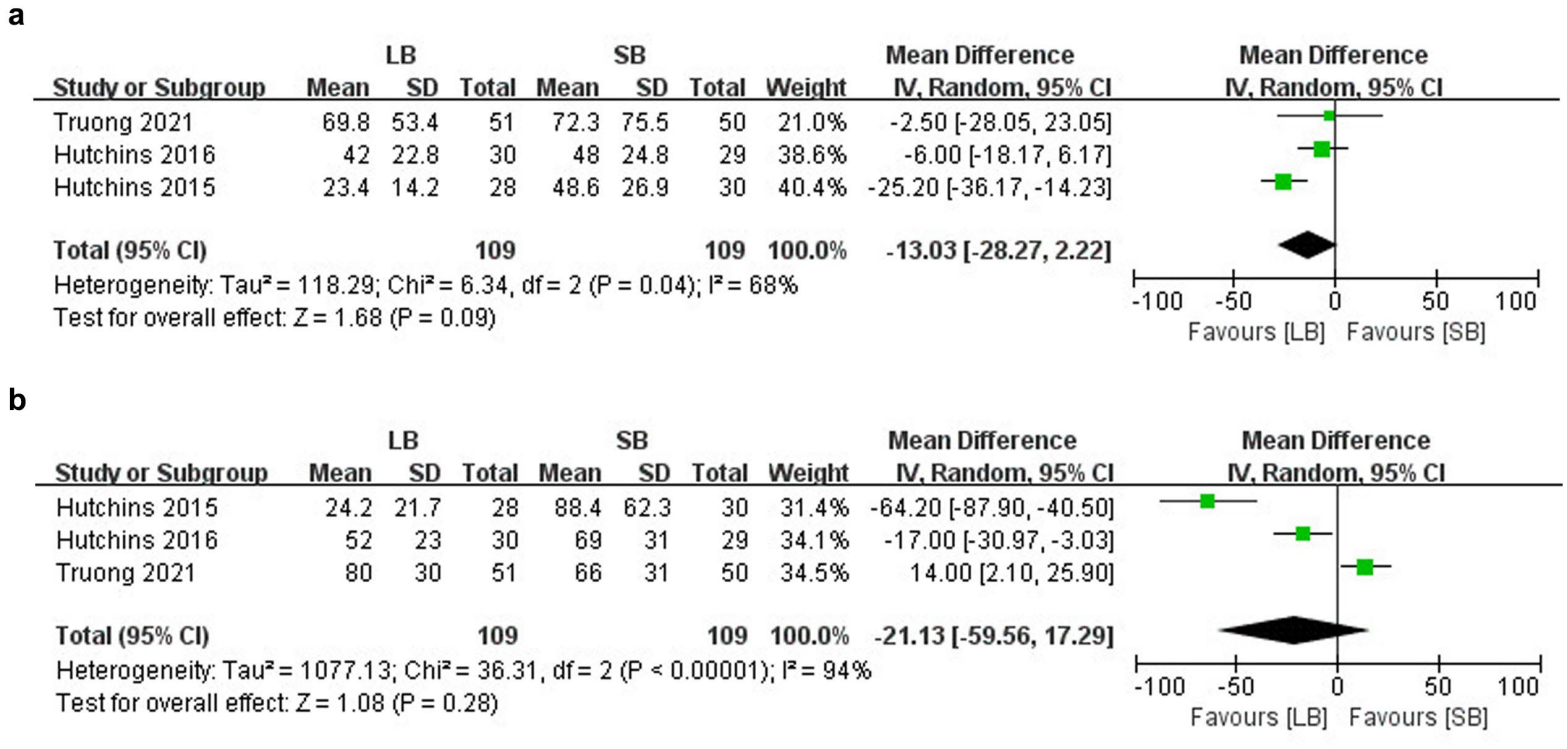
Forest plots of the cumulative MME comparing LB with SB+ adjuvants. **(a)** Within 2 days after surgery. **(b)** Within 3 days after surgery.

Three trials (*n* = 318) (20–22) reported the cumulative MME within 2 and 3 days after surgery, and the results revealed no between-group differences (MD: −13.03 mg, 95%CI: −28.27 to 2.22, *p* = 0.09, *I*^2^ = 68%; MD: −21.23 mg, 95%CI: −59.56 to 17.29, *p* = 0.28, *I*^2^ = 94%) (Figures 6a,b).

##### Average pain score

Only one trial (22) reported average pain scores. There were no between-group differences on day 1 or day 2 after surgery, but the pain score was significantly reduced on day 3 with liposomal bupivacaine (*p* = 0.004).

Time to first rescue: One trial (20) revealed no between-group differences.

Time to first passage of flatus: One trial (22) revealed no between-group differences.

Time to first ambulation: One trial (22) revealed no between-group differences.

PONV: Three trials (*n* = 218) (20–22) showed a significant reduction with liposomal bupivacaine (RR: 0.49, 95%CI: 0.32–0.75, *p* = 0.001, *I*^2^ = 0%) (Supplementary Figure 7).

Hospital stay: Three trials (*n* = 218) (20–22) showed no significant between-group differences (MD: −0.08 day, 95%CI: −0.47 to 0.31, *p* = 0.68, *I*^2^ = 92%) (Supplementary Figure 8).

### Quality of evidence according to the GRADE

The quality of evidence is presented in Table 2. All evidence comparing LB with SB or SB plus adjuvants was rated as low or very low.

**Table 2:** Quality of evidence according to the GRADE.

Outcomes	*N* participants (*N* studies)	RR	MD	95%CI	*p*-Value	*I* ^2^	Funnel plot	Egger’s test	Quality of evidence (GRADE)
LB vs. SB									
MME (within 1 day)	668 (7)	∕	−0.62	−1.13 to 0.11	0.02	49	Symmetrical	0.286	⊕⊕○○ (low)
MME (within 2 days)	816 (8)	∕	−0.53	−1.17 to 0.11	0.10	25	Symmetrical	0.306	⊕○○○ (very low)
MME (within 3 days)	521 (6)	∕	−10.34	−24.31 to 3.63	0.15	73	Symmetrical	0.810	⊕○○○ (low)
NRS (on day 1)	975 (7)	∕	−0.65	−1.03 to −0.28	0.006	58	Symmetrical	0.732	⊕⊕○○ (low)
NRS (on day 2)	1,123 (8)	∕	−0.40	−0.77 to −0.04	0.03	466	Symmetrical	0.425	⊕⊕○○ (low)
NRS (on day 3)	975 (7)	∕	−0.30	−0.48 to −0.13	0.0005	26	Symmetrical	0.154	⊕⊕○○ (low)
Time to first rescue	344 (4)	∕	5.39	0.06 to 10.72	0.05	78	Symmetrical	0.320	⊕○○○ (low)
Time to first passage of flatus	267 (3)	∕	−0.23	−0.35 to −0.11	0.0002	81	Symmetrical	∕	⊕⊕○○ (low)
Time to first ambulation	537 (5)	∕	−0.03	−0.07 to 0.00	0.08	95	Symmetrical	0.560	⊕○○○ (very low)
Local anesthetic-related toxicity	543 (4)	0.84		0.46–1.54	0.57	0	Symmetrical	0.321	⊕⊕○○ (low)
Hospital stay PONV	1,124 (9)	∕	0.11	−0.30 to 0.07	0.23	95	Symmetrical	0.639	⊕○○○ (very low)
	596 (5)	0.67	∕	0.43 to 1.04	0.07	0	Symmetrical	0.103	⊕○○○ (very low)
Liposomal bupivacaine vs. standard bupivacaine plus adjuvants									
MME (within 1 day)	318 (3)	∕	−6.89	−17.82 to 4.04	0.22	70	∕	∕	⊕○○○ (very low)
MME (within 2 days)	318 (3)	∕	−13.03	−28.27 to 2.22	0.09	68	∕	∕	⊕○○○ (very low)
MME (within 3 days)	318 (3)	∕	−21.23	−59.56 to 17.29	0.28	94	∕	∕	⊕○○○ (very low)
Hospital stay	318 (3)	∕	−0.08	−0.47 to 0.31	0.68	92	∕	∕	⊕○○○ (very low)
PONV	218 (3)	0.48	∕	0.32–0.74	0.0009	0	∕	∕	⊕⊕○○ (low)

MME, morphine milligram equivalent; NRS, numeric rating scale; PONV, postoperative nausea and vomiting.

## Discussion

Our meta-analysis demonstrates that LB can reduce cumulative opioid consumption within the first postoperative day and alleviate pain within the 3-day postoperative period compared to SB, but the extent of opioid reduction and pain relief is relatively modest, and the quality of evidence is low to very low.

Current multimodal analgesia approaches have diminished overall pain severity, with nonsteroidal anti-inflammatory drugs (NSAIDs) typically managing mild to moderate pain and opioids reserved for severe cases (23). In minor procedures such as hernia repair or hysterectomy, where postoperative pain is typically brief and mild, LB’s extended duration might offer limited advantage. Furthermore, while opioid consumption was converted to the MME, NSAID use was not quantified, potentially diminishing the clinical significance of comparisons based on opioid consumption. Our meta-analysis showed that LB slightly reduced cumulative opioid consumption within 1 day after surgery (MME of 0.62 mg) but had no effect on opioid use over 2–3 days after surgery. Several meta-analyses have reported that LB does not reduce postoperative opioid consumption compared to traditional local anesthetics when used for local infiltration (24), interscalene nerve block (25), or periarticular injection (26). Based on the very low to low quality of evidence, our results indicate that LB provides only a very limited benefit over SB in reducing opioid consumption in TAP blocks.

Postoperative pain remains a major concern for patients. Although it is subjective rather than objective, pain can negatively impact recovery, potentially increasing the risk of opioid use and dependence. The pooled results revealed that LB in TAP blocks significantly reduced pain scores on postoperative days 1–3 compared to SB. In our meta-analysis, LB reduced pain scores with an MD of 0.65 on day 1, 0.40 on day 2, and 0.30 on day 3 after surgery, in line with the findings of a previous meta-analysis comparing LB with traditional local anesthetics for peripheral nerve and field blocks across various surgeries (27). However, in a recent meta-analysis comparing LB with non-liposomal local anesthetics in brachial plexus blocks, an MD of 1 on the NRS (0–10) was considered the minimal clinically important difference in pain relief (28). Based on the low confidence level of evidence, it seems that LB provides only very limited postoperative pain relief in TAP blocks compared to SB. Several other meta-analyses also showed that LB had no advantage over traditional long-acting local anesthetics in periarticular injections for postoperative pain relief (29, 30).

Adjuvants to SB (e.g., adrenaline, morphine, dexmedetomidine, or dexamethasone) in TAP blocks improve postoperative analgesia and reduce opioid consumption. Our pooled analysis of three studies showed no superiority of LB over SB plus adjuvants. Since adjuvants can extend the block duration of SB, further research is needed to compare the efficacy of LB with SB plus adjuvants (31, 32).

This meta-analysis has several limitations. First, the included studies involved diverse surgical procedures and varied in local anesthetic concentration, volume, and TAP block technique (ultrasound-guided vs. laparoscopic-guided). We conducted meta-regression and did not identify any of the covariates (sample size, age, LB concentration and volume, surgical type, TAP methods, or publication year) as a significant moderator of the primary outcome. Second, few studies reported outcomes such as time to first rescue, time to first ambulation, or adverse events and toxicity related to local anesthetics. LB, as a novel drug formulation used in TAP blocks, requires further investigation to establish its clinical safety. Third, only a limited number of studies compared LB with SB plus adjuvants—two studies used adrenaline as an adjuvant (20, 21), and one study used both dexamethasone and adrenaline (22). The pooled results suggested non-inferiority between the two groups. Given that LB is relatively expensive, the cost-effectiveness of the drug for TAP blocks and its pain relief and adverse event profile compared to SB plus adjuvants should be evaluated in large-scale RCTs.

In summary, this meta-analysis demonstrates that liposomal bupivacaine used in TAP blocks can provide only very limited opioid reduction and pain relief after surgery compared to standard bupivacaine. In addition, liposomal bupivacaine showed no superiority over standard bupivacaine plus adjuvants in postoperative outcomes. Given the very low to low quality of evidence, large-scale RCTs are required to validate our findings.

## Supplementary material

SUPPLEMENTARY FIGURE 1 Forest plot of the time to the first rescue comparing LB with SB.

SUPPLEMENTARY FIGURE 2 Forest plot of the time to first passage of flatus comparing LB with SB.

SUPPLEMENTARY FIGURE 3 Forest plot of the time to first ambulation comparing LB with SB.

SUPPLEMENTARY FIGURE 4 Forest plot of PONV comparing LB with SB.

SUPPLEMENTARY FIGURE 5 Forest plot of Local anesthetic-related toxicity or adverse events comparing LB with SB.

SUPPLEMENTARY FIGURE 6 Forest plot of hospital stay comparing LB with SB.

SUPPLEMENTARY FIGURE 7 Forest plot of PONV comparing LB with SB+ adjuvants.

SUPPLEMENTARY FIGURE 8 Forest plot of hospital stay comparing LB with SB+ adjuvants.
