# Supplementary figures and images for: Efficacy of liposome bupivacaine in transversus abdominis plane blocks for postoperative analgesia: a systematic review and meta-analysis
Source: Front Med (Lausanne). 2026 May 19;13:1803767. doi: 10.3389/fmed.2026.1803767 (PMC13227398; doi:10.3389/fmed.2026.1803767)

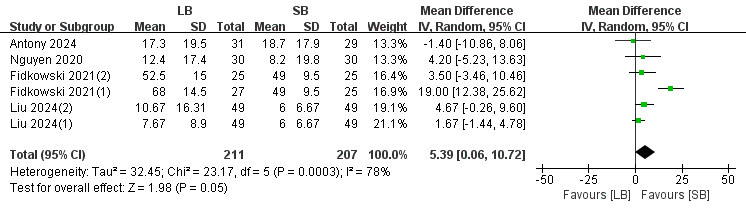

Supplement: SUPPLEMENTARY FIGURE 1 — Forest plot of the time to the first rescue comparing LB with SB. [file Image_1.jpeg]

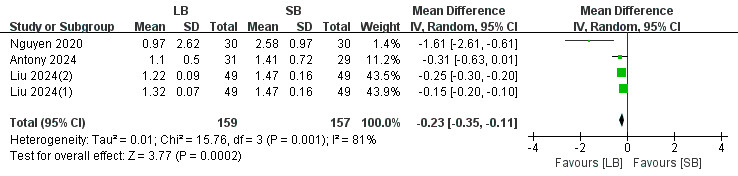

Supplement: SUPPLEMENTARY FIGURE 2 — Forest plot of the time to first passage of flatus comparing LB with SB. [file Image_2.jpeg]

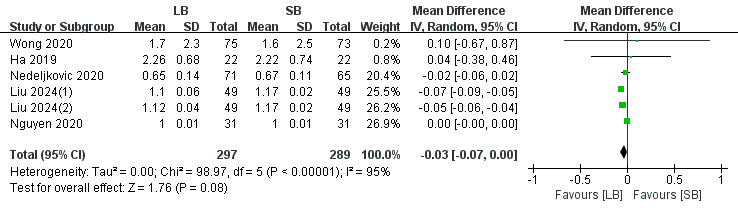

Supplement: SUPPLEMENTARY FIGURE 3 — Forest plot of the time to first ambulation comparing LB with SB. [file Image_3.jpeg]

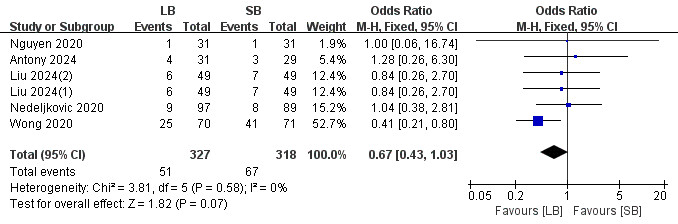

Supplement: SUPPLEMENTARY FIGURE 4 — Forest plot of PONV comparing LB with SB. [file Image_4.jpeg]

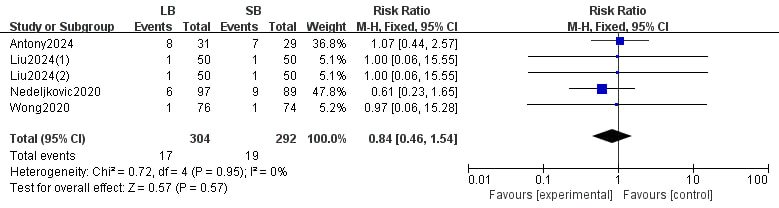

Supplement: SUPPLEMENTARY FIGURE 5 — Forest plot of Local anesthetic-related toxicity or adverse events comparing LB with SB. [file Image_5.jpeg]

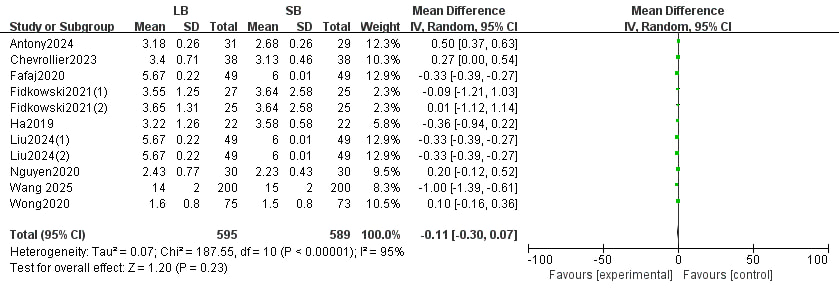

Supplement: SUPPLEMENTARY FIGURE 6 — Forest plot of hospital stay comparing LB with SB. [file Image_6.jpeg]

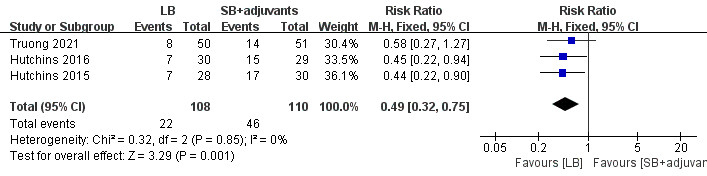

Supplement: SUPPLEMENTARY FIGURE 7 — Forest plot of PONV comparing LB with SB+ adjuvants. [file Image_7.jpeg]

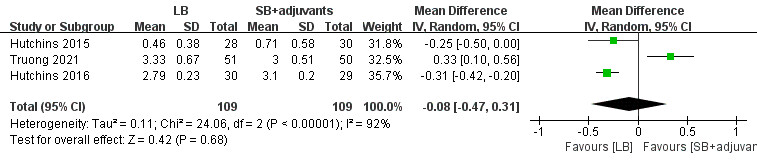

Supplement: SUPPLEMENTARY FIGURE 8 — Forest plot of hospital stay comparing LB with SB+ adjuvants. [file Image_8.jpeg]
