# Supplementary material for: Efficacy of liposome bupivacaine in transversus abdominis plane blocks for postoperative analgesia: a systematic review and meta-analysis
Source: Front Med (Lausanne). 2026 May 19;13:1803767. doi: 10.3389/fmed.2026.1803767 (PMC13227398; doi:10.3389/fmed.2026.1803767)
Supplement: Supplementary file 9 [file Table_1.docx]

Pubmed

1. liposome bupivacaine[All fields]
2. liposomal bupivacaine[All fields]
3. bupivacaine liposome[All fields]
4. #1 OR #2 OR #3
5. bupIvacaine [All fields]
6. plane bupuvacaine [All fields]
7. standardized bupivacaine[All fields]
8. simple bupivacaine[All fields]
9. regular bupivacaine[All fields]
10. conventional bupivacaine[All fields]
11. standard bupivacaine[All fields]
12. #5 OR #6 OR #7 OR #8 OR #9 OR #10 OR #11 OR 12
13. transversus abdominis plane block[All fields]
14. TAP block[All fields]
15. TAP[All fields]
16. #14 OR #15 OR #16
17. randomized controlled clinical trial[ptyp]
18. randomized[ptyp]
19. randomized controlled trial as topic[MeSH Terms]
20. randomized controlled trial[All fields]
21. randomised controlled trial[All fields]
22. #18 OR #19 OR #20 OR #21 OR#22

24. NOT animal

25. 2000/01/01[Date-publication]: 2025/07/31[Date-publication]

26. #4 AND #13 AND #17 AND #23 AND #24 AND #25

Embase

(‘liposomal’/exp OR ‘liposome’/exp AND ‘bupivacaine’) OR ‘liposomal bupivacaine’ OR ‘bupivacaine liposome’ OR ‘exparel’

AND

‘bupivacaine’/exp OR ‘bupivacaine’

AND

(‘transversus abdominis plane block’/exp) OR ‘transversus abdominis plane block’ OR ‘TAP block’

AND

‘Clinical trial’ OR ‘randomized controlled trial’

AND

[2000-2025]/py

Cochrane library

1. MeSH descriptor:[bupivacaine] explode all trees
2. (bupivacaine) ti ab kw
3. #1 OR #2
4. MeSH descriptor:[liposomal bupivacaine] explode all trees
5. (Liposomal bupivacaine) ti ab kw
6. (Bupivacaine liposome) ti ab kw
7. #4 OR #5 OR #6
8. (transverses abdominis plane block) ab ti kw
9. (TAP block) ab ti kw
10. #8 OR #9

10.MeSH Descriptor:[ randomized controlled trial) explode all trees

11. (randomized controlled trial) ti ab kw

12. #10 OR #11

13. py=2000-2025

14. #3 AND #7 AND #10 AND #12 AND #13
