# Supplementary material for: Efficacy of liposome bupivacaine in transversus abdominis plane blocks for postoperative analgesia: a systematic review and meta-analysis
Source: Front Med (Lausanne). 2026 May 19;13:1803767. doi: 10.3389/fmed.2026.1803767 (PMC13227398; doi:10.3389/fmed.2026.1803767)
Supplement: Supplementary file 10 [file Table_2.doc]

Fixed-effects meta-regression Number of obs = 9

Method: Inverse-variance Model F(6,2) = 1.59

Prob > F = 0.4342

---------------------------------------------------------------------------------------------------------------------------------

Covariates effect size Coefficient Std. err. t P>|t| 95% CI

-------------+------------------------------------------------------------------------------------------------------------------

Sample size -.2514112 .2043831 -1.23 0.344 -1.130801 .6279785

Age -.3610233 .5715503 -0.63 0.592 -2.820206 2.098159

Volume 1.326226 1.46606 0.90 0.461 -4.981722 7.634173

Concentration -2586.963 6137.442 -0.42 0.714 -28994.24 23820.32

Publication Year 1.251163 3.002318 0.42 0.717 -11.66677 14.16909

Surgery Type -6.063902 13.22963 -0.46 0.692 -62.9864 50.8586

TAP Method 34.69306 33.62819 1.03 0.411 -109.9974 179.3835

---------------------------------------------------------------------------------------------------------------------------------
